# The tenure track employment system in colleges and universities in China: a scoping review of the Chinese literature

**DOI:** 10.3389/fpsyg.2023.1271110

**Published:** 2024-01-10

**Authors:** Xin Wang, Wen Li Wang

**Affiliations:** School of Humanities, Jiangnan University, Wuxi, China

**Keywords:** tenure track, employment system, higher education, scoping review, Chinese literature

## Abstract

Chinese higher education institutions have adopted a US-style tenure track system since the 1990s. This is an important reform aimed at modernizing China’s higher education system. In response, authors have begun to carry out close examination of the career system and analyse its implications in a national context (Republic of China). This study aims to present the key research themes, identify research gaps and offer recommendations from the increasing pool of Chinese-language literature on the tenure track system. A scoping review of Chinese language papers was conducted using the China National Knowledge Infrastructure (including the China Academic Journals Full-text Database, China Core Newspapers Full-text Database, China Doctoral Dissertations Full-text Database, China Masters’ Thesis Full-text Database, and China Yearbooks Full-text Database) (CNKI) database. Four major research themes were identified in Chinese discourse: (1) examining the tenure track system, (2) providing suggestions for better adaptation of the tenure track system in the Chinese context, (3) analysing the negative effects of the tenure track system, and (4) analysing the positive effects of the tenure track system. Generally, authors were concerned with the adaptation and cultivation of the US-originated tenure track system in the Chinese context and emphasized the importance of acknowledging its perceived negative influences on early-career scholars who have not received adequate attention. Overall, the authors demonstrate increasing interest in the tenure track system in China, and the literature is of variable quality. Further empirical studies are needed to analyse, evaluate and guide future improvement of the career system in the Chinese context in practice.

## 1 Introduction

The tenure track system refers to a faculty employment system in which a university selects outstanding faculty who meet the requirements after a certain probationary period and grants them tenured employment with a number of institutional and legal rights, including ‘academic freedom’ (Herbert and Tienari, 2013). Tenure was created to protect academic staff members and guarantee them academic freedom so they could continue to teach and research as they best saw fit without fear of any repercussions (Miller et al., 2011). Typically, this means that academic staff members in a tenure track appointment can only be dismissed for grave misconduct.

The tenure track employment system originated in the US and became common in US universities in the last century (Herbert and Tienari, 2013). Academic staff members are expected to work in a period of probation before undergoing a review to determine whether they should be awarded a tenure track professorship. Along with US models of higher education, the concepts and values underpinning the US-style tenure track system were exported across the world (Engwall, 2007) as a response to the increased competition for talent and the declining attractiveness of an academic career which has been in focus in many countries (Henningsson et al., 2018). In 1994, Tsinghua University took the lead in introducing this system into China, and many Chinese universities have followed since.

In recent years, the tenure track system has aroused many concerns and mixed reactions in China, and authors have begun to recognize the importance of the re-examination of the tenure-track system in the Chinese context to better suit local higher education institutions. Hundreds of Chinese-language papers have been published on tenure-track systems, examining tenure-track system-related topics from different perspectives. To understand the scope of Chinese tenure-track-related research, this study reviews and summarizes the Chinese literature on the system in a national context (People’s Republic of China). We focus on the publications on the tenure track employment system in Chinese language for three reasons: first, past reviews of the tenure track employment system have examined publications in English (Nakao Pietilä and Yano, 2006; Khan and Jabeen, 2011; Pietilä, 2015; Henningsson et al., 2018; Pietilä, 2019; Castellacci and Viñas-Bardolet, 2020; Guillaume and Apodaca, 2020; Wang et al., 2023; Yang et al., 2023). Second, in a global age, there is value in examining the tenure track employment system from an international perspective, and China in particular has increased its contributions to the study of the tenure track system as growing attention has been paid to it in China, with hundreds of Chinese-language papers have been published on the tenure track system recently. Third, no studies to date have specifically examined the publications on the tenure track employment system in Chinese language yet, and we believe it is important to understand the tenure track system in China not only because of the large volume of researchers and universities staff worked there but also the rapidly changing trends in internationalization of Chinese higher education.

This is a topic that has thus far received little attention. This study aims to identify, first, existing literature published on the tenure track system. Second, it followed Arksey and O’Malley (2005)’s scoping review framework to analyse these studies in terms of their research topics and results. Finally, an analysis of the literature is presented and discussed in the results and discussion sections.

## 2 Background

There have been two stages in the adaptation of the tenure track employment system in China: a trial period and a large-scale promotion period.

### 2.1 Trial period

In 1993, Tsinghua University started to explore the reform of its employment system and put forward a “promote or leave” implementation plan. Tsinghua University was established in 1911, originally under the name “Tsing Hua Imperial College.” The school was renamed “Tsing Hua College” in 1912. The university section was founded in 1925 with 21 schools and 59 departments. As one of China’s leading universities, it took the lead to start reforming the traditional employment system with the intention of optimizing the faculty through the last elimination system and realizing the circulation of talent as well as enhancing its research and teaching quality.

In 1994, the “promote or leave” policy was formally implemented at Tsinghua University. It stipulates that those who cannot be promoted to the intermediate level within 3 years after reaching the tenure of the junior position or those who cannot be promoted to the deputy senior level within 5 years of reaching the tenure of the intermediate position will be transferred or dismissed from their post. It can be seen that Tsinghua University’s “promotion or leave” system was relatively mild at that time. It did not implement a one-size-fits-all approach, and there is a “non-promotion but transfer” plan for individuals to choose.

In 1999, Tsinghua University implemented an employment system that combines tenure track and tenured employment, institutionalizing the “promotion or leave” system. The new system emphasizes that new recruits should be employed on a continuous contract basis (Qian, 2013). It stipulates a minimum of two appointment periods for junior positions and a maximum of three periods for intermediate positions. If they cannot be promoted to a first-level position, they will not be reappointed. Associate professors and above can be employed for tenure tracks after one to two appointment periods. In the same year, Tsinghua University canceled the system of a qualification certificate for newly hired teachers and replaced it with a letter of appointment with a clear employment period, which is synchronized with the contract employment period. From 1998 to 2002, teachers whose employment contracts were not renewed accounted for 10.8% of the total number of signed appointments. To date, the contract system has gradually replaced the traditional employment system (also known as the “iron rice bowl”) and has been promoted at Tsinghua University (Beijing Evening News, 2014).

Later, in 2003, the reform of Peking University’s employment system was considered to be the beginning of the implementation of the tenure track system in Chinese universities. Originating as the Imperial University of Peking in 1898, Peking University was China’s first national comprehensive university and one of China’s most prestigious and influential universities. It has 55 schools and departments, 60 research entities, and 10 affiliated hospitals. At Peking University, associate professors have 2 opportunities to apply for the title of professor after 5 years of service. If the first application is unsuccessful, the second application must be made 1 year later; if the second application is also unsuccessful, those who are in a fixed employment period will not be renewed to their original position. Some academic staff from the Department of Humanities and Social Sciences argued that the assessment criteria were not applicable for the humanities and social sciences disciplines, and the tenure track system encountered greater resistance at Peking University at that time (Li and Zhao, 2016).

Fudan University started to adopt the tenure track system in 2007. Fudan University, which was established in 1905 as Fudan Public School, is also one of the leading universities in China. During the process of implementation, it was discovered that this system has drawbacks, such as quantifying academic research. The pressure to publish in peer-reviewed journals is likely to make early-career academics compromise research quality, turning academics into an arena for grandiose comparisons. In response to this concern, in 2010, Fudan University began to pilot the “academic masterpiece” system, and it has been implemented throughout the university since 2012. As the name suggests, the central evaluation principle of this system is the influence of academic achievements, that is, quality, not quantity (Beijing Evening News, 2014). This action can be seen as an important action made by officials to improve the tenure-track system in Chinese higher education institutions.

### 2.2 Large-scale promotion period

In 2014, the comprehensive reform plan of Tsinghua and Peking University was approved. Teaching posts in the tenure track system include 3 levels: professor, associate professor and assistant professor. Professors are tenured posts, associate professors can be tenured posts or tenure track posts, and assistant professors are tenure track posts.

After approval, in 2014, Tongji University launched the tenure-track system for newly recruited teachers, and in 2015, Wuhan University issued the document “Trial Measures for the Appointment System for Newly Selected Teachers of Wuhan University” and implemented the “tenure track period” system. Later, other colleges and universities in China followed up. At present, 34 out of the 39 “985” project colleges and universities in China have implemented the tenure track employment system, and most of the 211 project colleges and universities (“Project 211” refers to the construction of approximately 100 colleges and universities and a number of key disciplines in the 21st century.) and some of the research-leading universities in each individual province have also implemented the tenure track employment system. The period of implementation of the tenure track system in various colleges and universities was seen as the “large-scale promotion period.” Generally, most colleges and universities set up an assessment period for recruits of 5–7 years, although the tenure track employment system is more complex in a few universities.

The tenure-track system has played an important role in China’s higher education development over its nearly 30-year history. It can optimize the structure of the teaching staff, break the dilemma of faculty inbreeding, improve the efficiency of university organisation activities, establish academic systems and norms that are in line with the international community, and weaken the official-centric tendency within the university (Huang, 2020). The tenure-track system breaks through the traditional personnel management system by nurturing talent qualified to work both at the top and at the grassroots level. Additionally, it plays a significant part in fostering university teachers’ capacity for scientific research and works well to support their academic output. The tenure-track system has altered domestic colleges’ issues with nepotism, inbreeding, and teachers who procrastinate in their positions for an extended period when hiring new employees.

However, lately, some academics have started to draw attention to the fact that the tenure-track system also has a complex and multifaceted impact on the growth of China’s higher education. Numerous papers have started to examine the detrimental effects of the non-promotion or leaving system, covering everything from the macrolevel applicability of system transplantation to the microlevel long-term development of new teachers (Zhao, 2014; Zhang, 2018; Zhang and Chen, 2018; Liu, 2021). There is not yet a thorough analysis of the literature on this subject, even though more academics are becoming interested in it. We believe it is important to understand the impacts of the tenure track employment system in universities and colleges in China not only because of the volume of young academics produced and worked there but also for the future development of higher education worldwide.

To our knowledge, this is the first scoping review of published literature on the tenure track employment system in colleges and universities in China. As a scoping review is good at mapping “the key concepts underpinning a research area” that is “complex or has not been reviewed comprehensively before” (Mays et al., 2001) p. 194, this study aims to provide a scoping review of the available research in the Chinese database to fill the gap in the current literature and to assist the development of evidence-based research in the future.

## 3 Methods

Scoping review was employed in this study. As a relatively new approach, scoping review has become an increasingly popular research approach for synthesising research evidence. So far, no universal study definition or definitive procedure has been established for this method, and we adopted Arksey and O’Malley (2005)’s scoping review framework in this research. Compared to a full systematic literature review, a scoping review does not focus on appraising the quality of the included papers (Arksey and O’Malley’s, 2005); consequently, it provides a descriptive account of available research to form a map, which includes the volume, nature, and characteristics of the primary research for the targeted research area in a relatively short space of time. The scoping framework we used to guide this review comprises five stages, which will be presented below.

### 3.1 Stage 1: identifying the research question

Similar to a fully systematic review, the first stage of performing a scoping review is to identify the main research question or purpose of the review. Our research purpose was to explore what is known about the tenure track employment system in colleges and universities in China. We are aware that “the tenure track employment system” was not the only term that people used in different contexts; therefore, we have to keep in mind that other terms might emerge during our research process, and those studies that look at the tenure track employment system but used different key words should also be included in this review. For example, many authors also used “up or out” to refer to the system.

### 3.2 Stage 2: identifying relevant studies

The second stage refers to the identification of relevant studies. As indicated at the beginning of this section, a scoping review focuses on providing a comprehensive review of the available literature to answer the main research question. To do so, three members of our research team (JMY, LXZ, SWL) systematically reviewed the literature from 1 January 1995 to 31 December 2022 in the China National Knowledge Infrastructure (CNKI) database. CNKI is an extensive multi-disciplinary full-text database of over 3,500 journals published in China, with content beginning as early as 1906. It is the largest and the most authoritative source of China-based information resources in the world, covering various subjects such as politics, economics, humanity and social science, science and technology.

A search was conducted for articles on the tenure track employment system in Chinese universities and colleges. Chinese terms related to the tenure track employment system were used as keywords, including “准聘长聘制” (zhun pin chang pin, the tenure track employment system), “非升即走” (fei sheng ji zou, up or out) and “大学事业编制” (da xue shi ye bian zhi, officially budgeted posts in universities and colleges). We selected articles that presented issues or situations of mainland China, including works from non-Chinese authors who collected their data in China and published in Chinese. Studies focused on Hong Kong, Macao and Taiwan are not included in this research, as they might have a different code of conduct from that of mainland China due to political reasons.

### 3.3 Stage 3: study selection

**Figure 1:**
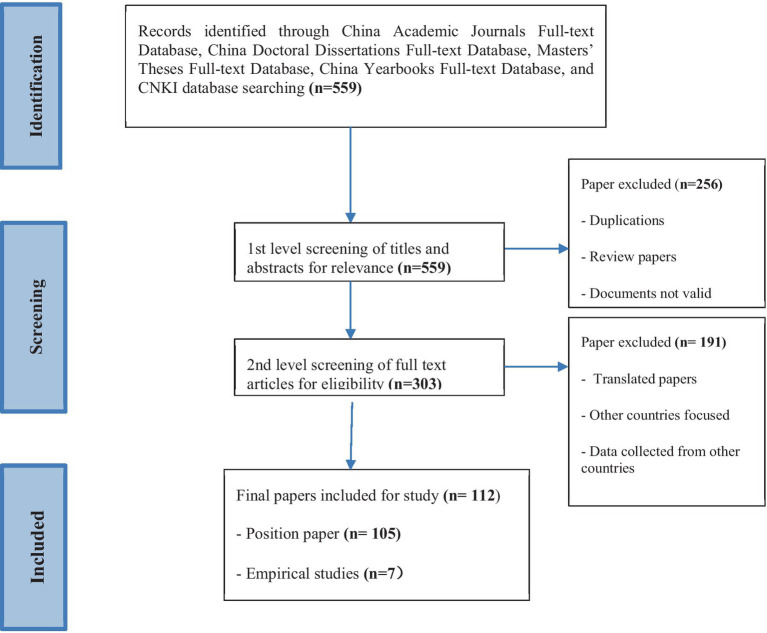
Overall flow diagram of the scoping literature search and selection.

The literature search was limited to the Chinese language during the past 28 years (1 January 1995–31 December 2022). The reason for the time limit was because the tenure track employment system was introduced into China in 1994. Articles directly matching or approximating these key words were identified. Titles and abstracts of articles were filtered for relevance to the tenure track employment system, and the full content was examined to determine suitability for inclusion in the review. We included all peer reviewed papers that addressed any aspect of the tenure track employment system in Chinese universities and colleges. An initial screening of 303 titles and abstracts was undertaken. Duplicates and any papers not meeting a broad inclusion/exclusion criterion were discarded. Forty-two papers went through a second level of screening of relevance. A further 34 papers were excluded, including articles translated from other languages and studies focused on countries other than mainland China (see Figure 1).

### 3.4 Stage 4: charting the data

The key items of information from the included articles were collated in a data chart form based on the research question, including author(s), year of publication, province where the study was conducted, research method(s), aims of the study, and research findings. Two reviewers (WX and WWL) independently tested the first draft of the data chart form among four random papers. Based on group discussions and reflections, the chart form was then revised. After approval by the team, it was then used to collate information from all the included studies narratively.

### 3.5 Stage 5: collating, summarizing and reporting the results

The data were analysed using qualitative thematic analysis (Hsieh and Shannon, 2005) with qualitative data analysis software NVivo (Bazeley, 2007), and the results are presented below in tables and in a descriptive manner. To ensure consistency, two reviewers (WX and WWL) each performed a thematic analysis of the literature independently and checked the results through comparison and discussion before the final results were approved by all reviewers.

## 4 Results

The initial database search identified 559 studies. After removing duplications, reviewed papers, invalid documents, translated papers, and papers that collected data from other countries, 112 peer-reviewed articles met the authors’ criteria for inclusion in this review.

### 4.1 General aspect of the literature

**Figure 2:**
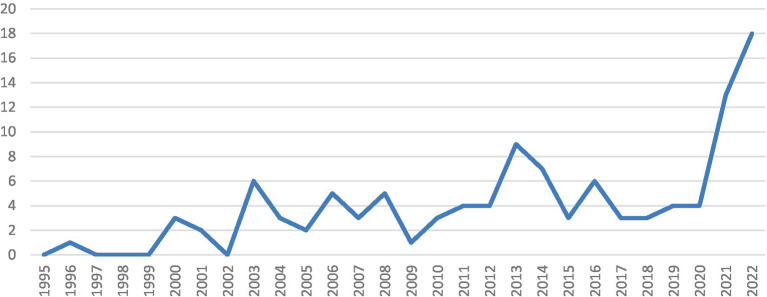
Number of publications on the tenure track employment system in Chinese from 1995 to 2022.

**Figure 3:**
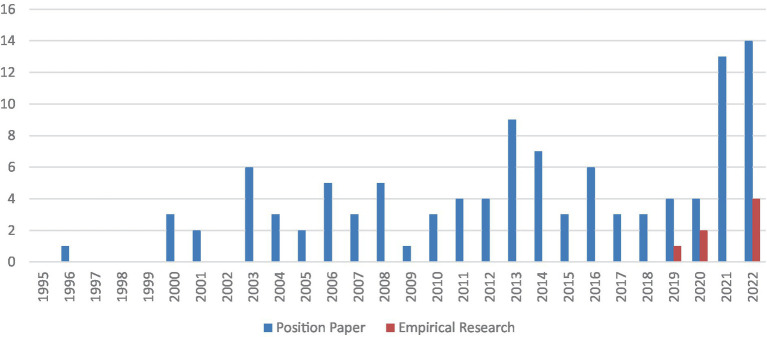
Number of publications: empirical studies vs. position papers.

The quantity of the tenure track employment system literature started to grow in 2000, and a distinct rise in quantity was observed in 2020 (see Figure 2). Of the literature reviewed, it is important to note that the majority of articles were position papers, with few empirical studies conducted. Of the 112 articles selected in this review, only 7 were empirical studies; the remaining 105 articles were position papers (see Figure 3). Four main themes were identified, including *introducing the tenure track employment system to the public, analysing the positive effects of the system, analysing the negative influences of the system, and providing suggestions for better adaptation of the system in the future in the Chinese context*. Note that a single article may address more than one theme.

### 4.2 Research themes

**Figure 4:**
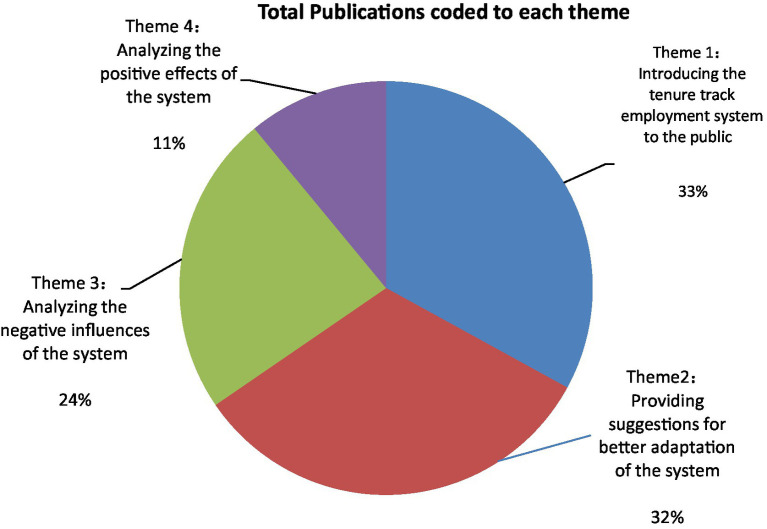
Publications coded to each theme.

**Figure 5:**
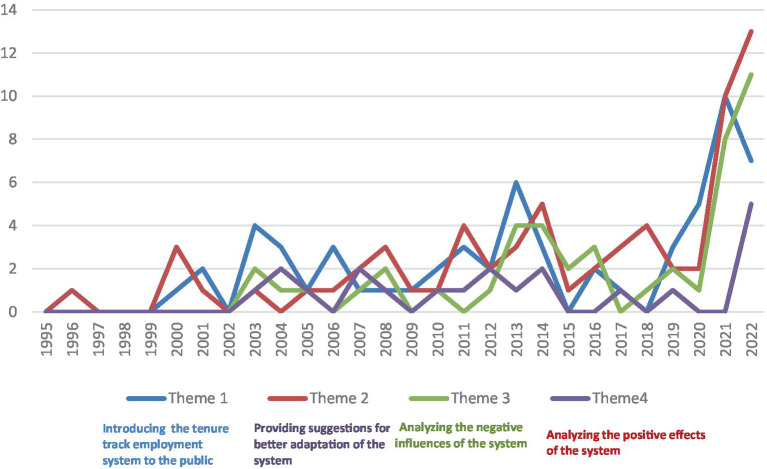
Publications coded to each theme by year.

Among the four identified themes, *introducing the tenure track employment system to the public (theme 1)* and *providing suggestions for better adaptation of the system (theme 2)* are the two most represented ones, followed by *analysing the negative effects (theme 3)* and *positive effects (theme 4) of the system* (see Figure 4). The content of the literature on the tenure track employment system also changed over time. Before 2020, articles dressed these four themes equally, and after 2020, more attention was placed on the latter two themes to analyse the negative effects of the system as well as to provide suggestions for better adaptation of the system in the Chinese context (see Figure 5).

#### 4.2.1 Theme 1: introducing the tenure track employment system to the public

The majority of the papers reviewed focused on introducing the tenure track employment system to the public (Wang and Zhang, 2004; Zhang, 2013; Wang, 2016; Xia, 2022). Some authors focused on changes that have been brought about to higher education institutions by this reform, including the ways in which teachers’ performances are measured (Zhong and Li, 2009; Xia, 2022), growing managerialism in higher education institutions that prioritizes efficiency and effectiveness (Zhao and Tian, 2003), and changes in universities’ organizational culture (Zhu et al., 2017).

Despite an interest in examining the tenure-track system in detail, many authors have also carried out comparative studies to uncover how the system was practiced differently in the US and in China from both the macro level perspective and the micro level perspective, with the purpose of developing a comparative view of it (Xu, 2014). Authors have suggested several areas for attention; for example, Chen and Wen (2020) argue that the screening rate in China is much higher than the international average rate. According to their research, over 60% of tenure-track candidates at many US institutions typically succeed, while in China, some universities introduced the tenure track system in the form of tenure track postdoctoral candidates, and only 25–30% of them could pass their assessments (Chen and Wen, 2020). Wang (2016) argues that, currently, there are no regulatory standards for the system in China, and in some universities, the probation period is either too short or too long to be effective in practice. Zhang et al. (2015) found that the flow of teachers in American colleges and universities is relatively frequent, and individuals who do not succeed in obtaining tenured teaching posts by the end of their contracts can still compete for a new position at other universities or move on to the industry to find a more suitable position. However, in China, those who have not passed their assessment typically have to accept administrative roles or find employment elsewhere. As most universities have an age limit for recruiting doctors (generally under the age of 35), those who were not promoted for the first time can only apply for colleges and universities from a lower ranking group.

#### 4.2.2 Theme 2: providing suggestions for better adaptation of the tenure-track system in the Chinese context

The second major theme discussed in the literature is *providing suggestions for better adaptation of the system in the Chinese context*. Sixty-three papers coded this theme and reported varied suggestions with the purpose of improving the local applicability of the system. Figure 5 shows that there is a distinct rise in the quantity of literature published under this theme starting in 2020, which made it the most represented theme in the reviewed literature after 2020.

This trend in the literature relates to current challenges in practicing the tenure track employment system in China. The system has been strong for academic publications and professional growth in young academics; however, recent social changes have drawn renewed attention to the influences of the system. In recent years, there have been frequent cases of teachers who have not been promoted to professional titles being dismissed or transferred, which has become a complex social phenomenon. Some authors argue that simply borrowing the system from the US was not a good option, as China has a different sociocultural background and political system, which may create barriers to its acclimatization (Zhong and Li, 2009; Zhu et al., 2017; Wang and Li, 2021; Wang and Pang, 2022). Some studies (Zhu, 2021a) argue that Chinese public colleges and universities have a long tradition of being identified as highly interfered with by the executive power of the state and academic positions in universities and colleagues have been treated as permanent officially budgeted positions. To replace the traditional permanent employment system with the “up or out” system in the Chinese context, the key issue to success is to understand the logic or mechanism of “commitment-threat/promise” that makes “up or out” work well in Western countries (Zhu, 2021b).

Various suggestions were provided for improvement from the reviewed literature. For example, Tian and Jiang (2022) highlight the importance of nurturing a supporting social culture for the “up or out” system by which “failure” could be treated as a common and acceptable option, as in traditional Chinese culture, “to conquer or to die” (不成功便成仁) is emphasized by Confucianism, and teachers who failed to get promoted under the tenure track employment system could be seen as ‘losers’. Other authors note the importance of improving the evaluation process, as existing evaluation criteria for promotion emphasize the quantity of one’s research outputs while overlooking research quality, influences of, and contributions to society (Wang and Pang, 2022). Wang (2021) and Fang (2019) call for a more open and transparent evaluation system and consistent criteria, as they argue that decisions to renew contracts or grant tenure are sometimes based on personal connections instead of academic merits; other researchers call upon establishing independent professional associations for evaluation to ensure fair treatment for all (Zhang and Ku, 2001). Huang (2021) suggests that the assessment period of the tenure track system in China should be standardized to 6 years. He argues that the current length of the assessment period in some institutions ranges from 3 to 9 years, which could be less effective in selecting excellent young researchers. Other articles have examined the current conditions of young academics and argue that it is essential to build a strong mentorship system as well as to improve the remuneration package for tenure-track candidates by providing them with necessary support when experiencing critical or challenging periods (for example, illness, childbirth, etc.), to support junior faculty members on their path to tenure (Wang and Pang, 2022). Overall, the authors express a pressing need to improve the tenure track system with more normalization as well as greater flexibility to better suit the Chinese context.

#### 4.2.3 Theme 3: analysing the negative influences of the tenure-track system

The third theme focuses on *analysing the negative influences of the tenure track system in China.* Similar to the previous theme, this theme also shows a distinct rise in the quantity of published literature since 2020, making it the second most represented theme in the reviewed literature (see Figure 5). This trend in the literature relates to recent social changes as well as significant social events related to the tenure track employment system in China in recent years that have sent waves through China’s research community (e.g., the killing of a mathematics faculty member on a Shanghai campus in 2021).

According to the literature, Chinese academics are negatively influenced after the implementation of the tenure-track system in the following areas: research performance, teaching performance, and personal lives. For example, Fang (2019) notes that to meet assessment standards, young scholars on tenure-track contracts tend to focus on research outputs that are “quantifiable” against recognized standards and give up research topics that require more time to explore regardless of their academic significance. Tian and Jiang (2022) also reported that, pushed by publication obsession, researchers are more likely to conform with existing theories or approaches rather than carry out research into innovation, which will eventually hinder the research performance of the whole group.

Apart from research performance, academic staff members’ teaching performance is also negatively affected. Huang and Fan (2015) reported that intense conflicts between teaching and research have been noticed, and academic staff prioritize research and perceive teaching as associated with low status. Early-career scholars have been reported as switching their work from teaching to research outputs, which can negatively affect teaching quality.

Finally, according to the reviewed literature, academic staff’s personal lives were also reported as negatively affected by the tenure-track system. Liu et al. (2012) find in their research that to meet contractual requirements, researchers generally report working for long hours under higher pressure, which could result in physical exhaustion and contribute to the development of work-related diseases such as insomnia and gastritis. Tian and Jiang (2022) also argue that the tenure-track system particularly disadvantages female professionals who have greater family responsibilities to fulfill. Ding and Hu (2018) find that some Chinese universities often overrecruit and push young researchers to produce many publications to enhance their international/national ranking as well as to attract more funding, and candidates who meet all their requirements for tenure are often not successful because their positions are limited. This continuous pressure of competition between colleagues could also negatively affect interpersonal relationships between academic staff and result in a sense of insecurity, anxiety and a feeling of powerlessness. Overall, the authors argue that the tenure-track system has caused various negative effects for some academic staff in terms of their research performance, teaching performance, and personal lives.

#### 4.2.4 Theme 4: analysing the positive effects of the tenure-track system

The last theme identified from the literature is *analysing the positive effects of the tenure-track system.*11% of the total reviewed literature is coded to this theme. Researchers have mainly discussed the positive influences brought about by the system from different perspectives. For example, Song (2007) argued that the tenure-track system aims to recruit all positions through open and fair processes instead of internal appointments; therefore, it is better than any of the other alternative systems previously used if it can be applied as intended in Chinese higher education institutions. Others also noticed that the system was designed to provide tenured academics with financial security and academic freedom to conduct research on their interests, which is helpful in nurturing a culture of innovation for academia.

One of the main reasons that theme four had the lowest percentage is because the tenure-track system adopted in China operated differently compared to that in the US. In the US, the tenure-track system usually does not have promotion criteria restrictions. Candidates generally can be promoted as long as they are able to meet the assessment standards. While in China, the tenure-track system introduced restrictions on top of the promotion criteria. Typically, there are a limited number or percentage for lecturers/assistant professors to be promoted even they all successfully met their assessment criteria. This leads to intense competition and have caused serious incidents in the past decades (as mentioned in the manuscript section 4.2.3, first paragraph). On September 28, 2020, the Communist party of China (CPC) central committee and the State Council issued an overall plan for deepening educational evaluation reform in the new ear, and set out new requirements for refining the educational assessment system in higher education institutions (CPC, 2020). In 2022, the Central Committee of the China Democratic Legal (CPPCC) initiated a proposal on implementing the “pre-employment system” of young teachers in universities and colleges to replace the existing tenure-track system (CPPCC, 2022). Some universities, including 6 research leading universities in China, have started to replace the tenure-track system with the “pre-employment system” according to the Tencent National News.^1^

## 5 Discussion

This scoping review of the tenure track system literature in the Chinese language provides important insights into the field. It showed an increase in the number and variety of papers on this topic over the past two decades. In recent years, following the frequent cases of teachers who failed on their path to tenure and significant social events related to the tenure-track employment system in China, the number of publications on the reviewed database increased, reflecting a rising concern with re-examining and improving the tenure-track system in Chinese content.

According to the review, this phenomenon of interest has been explored in both theoretical and empirical literature, with significant differences observed. Of the 112 articles reviewed from 1995–2022, 7 were empirical research articles, and the remaining 105 were position articles. One possible reason for a lack of empirical research on the subject might be that the tenure track employment system in many Chinese universities is not transparent, as argued by many authors, and the evaluation criteria are characterized by ambiguity, arbitrariness, and operability, making it difficult for researchers to obtain accurate data to carry out empirical studies. Another reason for such a lack is that theoretical reflection and reasoning still tend to be the favored research method compared to qualitative and quantitative research methods for Chinese authors. This echoes previous research that states that the number of empirical studies published in education in China is less than 15% of the total number of publications in the field (Yuan, 2017). This result reflects the fact that there is a crucial need to encourage and support research paradigm transformation in education research in China to produce more evidence-based empirical studies that can offer insights and solutions that target real problems that have emerged in practice.

This review revealed that in practice, the tenure track system has effectively functioned in only a small number of universities in China; however, the problem is not with the system itself but with its discretionary implementation at certain institutions. Although China has adopted the criteria of the tenure track system in theory, in practice, there are often large disparities among how these criteria are applied. The number of articles studying the negative effects of the tenure track employment system in China is significantly higher than that of articles studying the positive effects, suggesting a high dissatisfaction rate with the tenure track employment system and the perception that the tenure track employment system is unfriendly to young lecturers. According to the literature, the implementation of the tenure-track system has led to intense competition among universities in China, with an overemphasis on improving research performance at the expense of teachers’ personal development in some of the institutions investigated. This could be explained by the fact that the assessment criteria developed by universities do not truly reflect the values of the tenure-track system, as efficiency and effectiveness were overemphasized, threatening and suppressing academic and moral values traditionally embedded in higher education. During the process of producing measurable research output, some important elements are at risk of being overlooked, such as academic freedom, equalities, respect and trust for each other (Zhu, 2013).

The authors also suggested that in some universities, it is still more institution-led than faculty-controlled, and the appointment process lacks power checks and balances such as collective bargaining systems and “faculty consent mechanisms” from the formulation of appointment policies to the “interpretation” of assessment indicators to the “interpretation” of promotion rules. Teachers, as the implementers of reform, do not have many choices from the formulation of tenure policies to the “interpretation” of assessment indicators to the implementation and modification of promotion rules (Fang, 2019). In the absence of labour management, contract terms and promotion criteria are prone to “drift” and waver with changes in university rules. Researchers have also argued that the tenure-track system. Researchers have also argued that the tenure track system has been abused by some institutions to take advantage of young scholars through overrecruiting, and high tensions have been noticed over tenure, which contribute to the development of job-related physical and psychological diseases (Wang and Pang, 2022). By reviewing the literature, it can be learned that many universities in China did not comply with important regulations such as the *Labor Contract Law*, and some of the legitimate rights of university faculties are not effectively protected. This is likely to be one of the areas that need to be researched more in the future.

This scoping review also verbalized the need for both formal and informal support for tenure track candidates to better cope with pressures and other issues brought about by the system. Some researchers called for a strong mentorship system as well as to improve the remuneration package for tenure track candidates to offer them essential support during critical periods, while others highlighted the need to nurture a supporting social culture for the system by which “failure” could be treated as an acceptable consequence.

Overall, there is a need for further studies to assess and improve the tenure track system in China. The Chinese State Council has issued a plan to promote the development of ‘world-class universities and world-class disciplines’, which mainly targets its elite 985 and 211-project universities. Regardless of the negative influences discussed above, the tenure track employment system played a significant role in increasing the competitiveness and overall ranking of higher educational institutions. For example, Zhongshan University in Guangdong Province has attracted thousands of young talent to the university with a “generous” annual salary since 2017, and it has ranked second in the country in terms of the number of funded projects for the period of 2017–2022, and its international ranking keeps rising at the same time. The tenure-track system will continue to spread as China has targeted further enhancement of its educational strength and competitiveness in the global knowledge economy, and long-term planning and management of the tenure-track system will be necessary to promote a wider and better adaptation of the system.

There are some limitations of this review. Although all reviewed articles were publications listed in the academic database, the majority were position papers rather than empirical studies. It is difficult to draw a conclusive or comprehensive picture of the tenure-track system in China based on the limited empirical evidence available. However, it is possible that other studies of the tenure-track system in China exist but were published in other languages or were unpublished and consequently not covered in this review.

In general, there are more position papers and fewer empirical studies in the field of higher education research among Chinese scholars, which is related to the research paradigm preferred by Chinese scholars. Although empirical studies have received increasing attention in Chinese academia in recent years, the total number of empirical studies published in Chinese core academic journals remains low compared to other countries and provides limited guidance for education policies and practices in general as those recommendations offered by most of the position papers often involve grandiose plans and vague statements.

## 6 Conclusion

This scoping review of the tenure-track system literature in the Chinese language demonstrates a growing concern with adopting and improving the US tenure-track system in Chinese higher education institutions in practice. Authors are interested in examining the tenure-track system as well as providing suggestions for improvement in the Chinese context. There has also been re-examination of the positive and negative influences of the system. Continuing efforts to examine, evaluate and improve the system are necessary for a better adaptation of the tenure-track system in the Chinese context.

In the future, more empirical research on the tenure-track system is needed to have a closer examination of existing problems in the tenure-track system in Chinese higher education institutions to offer suggestions so that the tenure-track system could play a better and more positive role. Future research should also pay more attention to how to better localize the evaluation system of the tenure-track system, including establishing more detailed evaluation standards of professional titles, improving the flexibility of the system so it can better fit the traditional Chinese cultural context and contribute to the improvement of university staff efficiency. Meanwhile, as the current scoping review has provided rigorous and transparent results for mapping areas of the tenure-track system literature in the Chinese language, it could be used as a preliminary step to a full systematic review in this field in the future.

## Data availability statement

The raw data supporting the conclusions of this article will be made available by the authors, without undue reservation.

## Author contributions

XW: Conceptualization, Data curation, Formal analysis, Investigation, Methodology, Writing – original draft, Writing – review & editing, Funding acquisition. WW: Data curation, Validation, Resources, Writing – original draft.

## Footnotes

^1^http://new.qq.com
